# Artificial stimulus-response system capable of conscious response

**DOI:** 10.1126/sciadv.abe3996

**Published:** 2021-04-09

**Authors:** Seongchan Kim, Dong Gue Roe, Yoon Young Choi, Hwije Woo, Joongpill Park, Jong Ik Lee, Yongsuk Choi, Sae Byeok Jo, Moon Sung Kang, Young Jae Song, Sohee Jeong, Jeong Ho Cho

**Affiliations:** 1 SKKU Advanced Institute of Nanotechnology (SAINT), Sungkyunkwan University, Suwon 16419, Korea.; 2 School of Electrical and Electronic Engineering, Yonsei University, Seoul 03722, Korea.; 3 Department of Chemical and Biomolecular Engineering, Yonsei University, Seoul 03722, Korea.; 4 Department of Energy Science and Center for Artificial Atoms, Sungkyunkwan University, Suwon 16419, Korea.; 5 Department of Chemical and Biomolecular Engineering, Sogang University, Seoul 04107, Korea.; 6 Institute of Emergent Materials, Sogang University, Seoul 04107, Korea.

## Abstract

A stimulus-response system and conscious response enable humans to respond effectively to environmental changes and external stimuli. This paper presents an artificial stimulus-response system that is inspired by human conscious response and is capable of emulating it. The system is composed of an artificial visual receptor, artificial synapse, artificial neuron circuits, and actuator. By incorporating these artificial nervous components, a series of conscious response processes that markedly reduces response time as a result of learning from repeated stimuli are demonstrated. The proposed artificial stimulus-response system offers the promise of a new research field that would aid the development of artificial intelligence–based organs for patients with neurological disorders.

## Teaser

A successful emulation of human conscious responses is demonstrated through an artificial stimulus-response system.

## INTRODUCTION

Humans can recognize various stimuli and have a sophisticated reaction system to respond to them. A design of an artificial stimulus-response system and a device for using it have recently received research attention because such a system can contribute to improving the lives of humans with impaired nervous systems (*1*–*8*). Developments have been made to mimic the human stimulus-response mechanism and to apply them to living organisms (*9*–*15*). Recently, Lee group (*9*) invented an artificial afferent nervous system comprising pressure sensors, an organic ring oscillator, and a synaptic transistor. They demonstrated that the artificial nerve could be made compatible with living beings by being connected to the nerve of a cockroach and subsequent actuation of a leg muscle by stimulation. For artificial nervous systems mimicking the biological nervous system to be applicable to humans, they must have the characteristics of human responses (*16*–*22*). Human responses to external stimuli are classified into unconscious and conscious responses depending on, respectively, the absence or presence of awareness and ability to control the response. Unconscious responses such as knee reflexes or Pavlov’s dog experiment do not involve a conscious decision (*23*). In contrast, conscious responses are those that require a learning process by the cerebral cortex, such as an athlete’s starting reaction or catching a flying ball. This type of response can be controlled by learning from repeated stimuli and responses, and this learning modulates the synaptic connections and thereby optimizes the response (*18*–*22*, *24*). In this context, many researchers have dedicated efforts to the realization of artificial synapses as electronic devices (*25*–*32*). Recently, advanced artificial neural systems combined with stimulus sensors and actuators have been reported (*1*–*3*, *9*–*14*, *33*–*35*). For example, Lee group (*11*) successfully realized light-interactive actuation using an optoelectronic sensorimotor synapse and a neuromuscular system. However, these systems performed signal transmission based on unconscious response, but they did not completely mimic the biological stimulus-response system with conscious response. Simulation of the human stimulus-response system requires integration of neurons capable of conscious responses, stimulus detection, actuation, and control of synaptic connection strengthened by a learning process.

In this study, we developed an artificial stimulus-response system capable of conscious response. The developed system consists of a quantum dot (QD) photodiode, a retentive electric double layer (EDL) transistor, complementary metal-oxide semiconductor (CMOS)–based artificial neuron (AN) circuits, and a robot hand, which correspond to the visual receptor, synapse, neuron, and muscle, respectively. A vertical graphene/indium gallium zinc oxide (IGZO)/indium tin oxide (ITO) heterostructure was used as the base layer for both the artificial visual receptor (AVR) and the artificial synapse (AS). The photoresponse behavior of the AVR was induced via the photogating effect of the InP QD layer deposited onto graphene. The synaptic function of the system was successfully implemented by the modulation and retention of the graphene work function through control of ion movement in the retentive EDL. Furthermore, signal transmission was achieved using the retentive EDL in the AS and the CMOS neuron circuit. Last, artificial conscious response was generated by the integration of the robot hand with the above-listed components of the artificial nervous system, and the response time of the system was successfully controlled with far less circuit complexity than conventional all-CMOS–based system. In addition, because of the pulse-based operation, the lower energy consumption was achieved compared to all-CMOS–based circuit. On the basis of the findings of the present study, we expect that the realization of an artificial stimulus-response system will open a new chapter in research on organs with artificial intelligence that would be useful for the treatment of patients with neurological disorders.

## RESULTS

### Artificial stimulus-response system

**Fig. 1:**
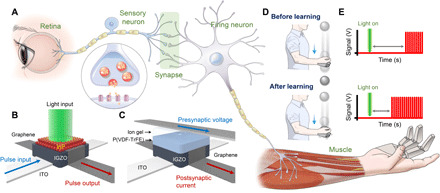
Artificial stimulus-response system. (**A**) Schematic illustration of biological stimulus-response system composed of retina, neurons, synapse, and muscle. (**B** and **C**) Schematic device structure of (B) AVR with InP QD layer and (C) AS with retentive EDL. (**D**) Schematic illustration of a human individual catching a ball successfully through learning. (**E**) Neural signals before and after learning process.

Figure 1A shows a schematic illustration of the biological stimulus-response system, which is composed of a retina, neurons, a synapse, and a muscle. First, the retina detects an external light stimulus and converts it into an electrical signal. Next, the sensory neuron sends an action potential to the synapse, which facilitates or depresses the signal. The firing neuron then analyzes and interprets the signal and delivers the integrated signal to the muscle. Last, the muscle performs an action as a response. This response process is analogous to that in our proposed artificial stimulus-response system, which consists of an AVR, AN circuits, an AS, and a robot hand. In this system, the AVR converts an external light stimulus into an electrical signal. Next, the sensory AN circuit transmits the electrical signal from the AVR to the AS, which performs integration of the sensory input. Then, the firing AN circuit generates appropriate responses to operate the robot hand. Figure 1 (B and C) illustrates the vertically stacked structures of the InP QD–based AVR and retentive EDL–based AS, respectively. The AVR and AS both have the graphene/IGZO/ITO heterostructure as the base layer, which serves as a platform for signal transmission (fig. S1). Note that the vertical structure provides a higher integration density than conventional lateral devices. The InP QD layer in the AVR is coated onto graphene, and this layer converts a light stimulus into an electrical signal by the photogating effect (*36*). For fabrication of the AS, poly(vinylidene fluoride-trifluoroethylene) [P(VDF-TrFE)] and an ion gel {a mixture of poly(vinylidene fluoride-*co*-hexafluoropropylene) [P(VDF-HFP)] and 1-ethyl-3-methylimidazolium bis(trifluoromethylsulfonyl)imide ([EMIM][TFSI]) ionic liquid} are coated to form the retentive EDL, which controls the synaptic connections through a learning process. Synaptic connections in a conscious response can be facilitated using the above described AVR, AS circuit, AN circuits, and robot hand. Figure 1D depicts the learning process, wherein through learning, a human individual is able to successfully catch a ball that starts to fall immediately after provision of light illumination as a stimulus. This successful catching is attributed to enhanced synaptic connections after learning and consequently, a faster response. Figure 1E shows the neural signals before and after the learning process. The decrease in the time interval between the start of illumination and the generation of the pulse train indicates a reduced response time, which is in agreement with the result depicted in Fig. 1D.

### Electrical properties of AS with retentive EDL

**Fig. 2:**
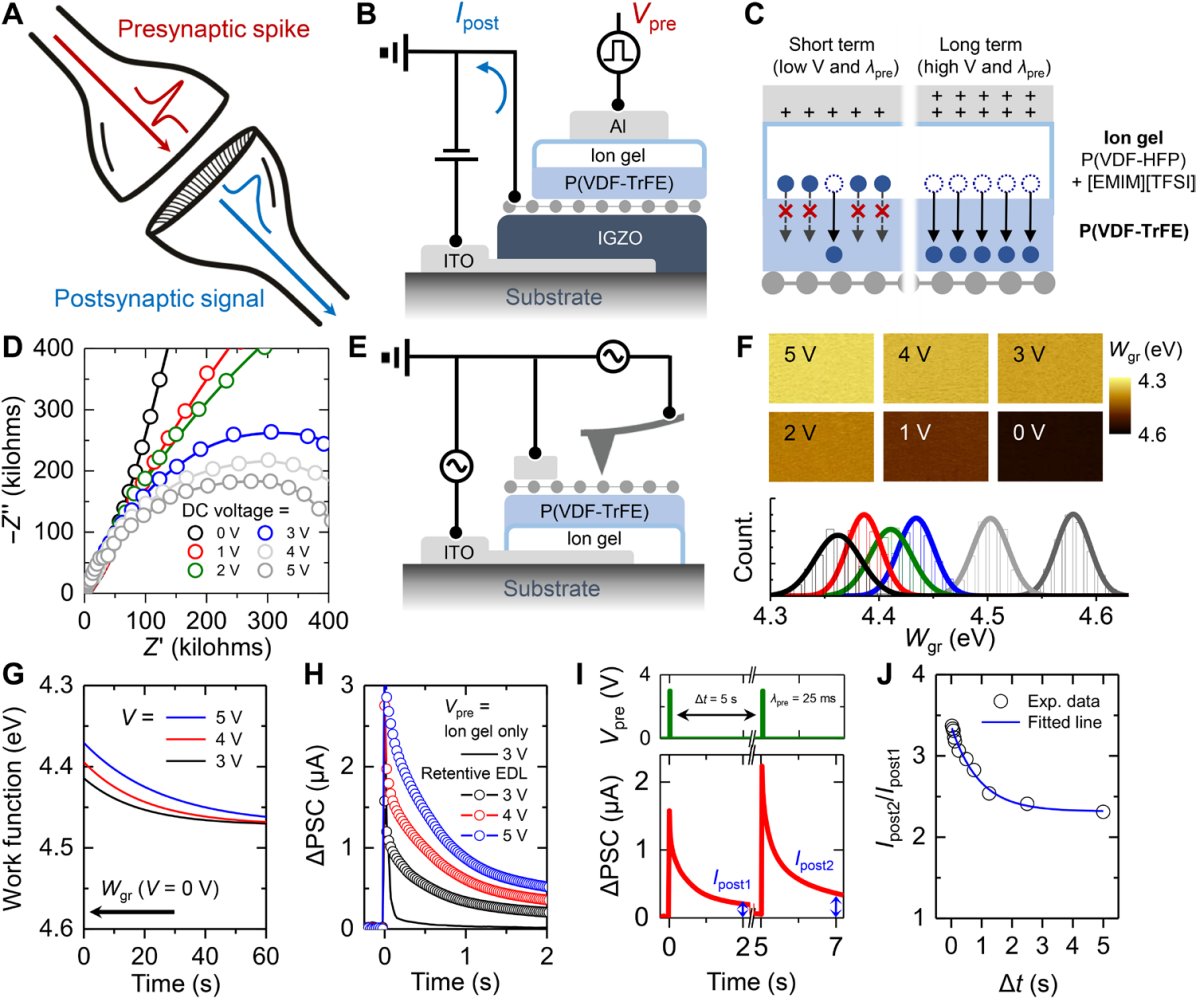
Electrical properties of AS with retentive EDL. (**A**) Schematic of biological synapse in stimulus-response system. (**B**) Cross-sectional schematic of AS based on vertically stacked graphene/IGZO/ITO heterostructure and retentive EDL. (**C**) Schematic of Al/retentive EDL/graphene heterostructure, describing the working mechanism of AS. (**D**) Nyquist plot of retentive EDL at various voltages. (**E**) Schematic of Kelvin probe force microscopy (KPFM) measurement setup. (**F**) KPFM images and histograms of *W*_gr_ at various voltages. (**G**) Retention properties of *W*_gr_ as determined by KPFM at various voltages. (**H**) Comparison of postsynaptic current (PSC) behaviors with two different gate dielectric layers (ion gel only and retentive EDL). (**I**) PSC triggered by two *V*_pre_ pulses applied with time interval of Δ*t*. *I*_post1_ and *I*_post2_ are the increased PSC value at 2 s after application of the first pulse and the second (consecutive) pulse, respectively. (**J**) Plot of *I*_post2_/*I*_post1_ as a function of Δ*t*.

The retentive EDL–based AS was inspired by the biological synapse in the stimulus-response system (Fig. 2A). The biological synapse transfers an action potential from the presynaptic terminal to the postsynaptic terminal. Synaptic connections in conscious response can be modulated by the presynaptic spike input (conscious decision), which causes a change in the postsynaptic signal (*20*). The postsynaptic signals show different trends depending on the degree to which synaptic connections are maintained. Short-term plasticity (STP) is achieved through either a temporary increase or a temporary decrease in the synaptic connections by the application of a single spike or infrequent spikes. In contrast, application of repeated presynaptic spikes causes a long-term change in the synaptic connections. A long-lasting increase in synaptic connections is referred to as long-term potentiation (LTP), and a long-lasting decrease is referred to as long-term depression (LTD).

Figure 2B shows a cross-sectional schematic of the vertically stacked AS with the graphene/IGZO/ITO heterostructure. First, the ITO drain electrode was sputtered onto a glass substrate, which was followed by the deposition of the IGZO layer. Then, monolayer graphene (fig. S2), which served as a postsynaptic terminal, was transferred onto the IGZO layer; this resulted in the formation of a Schottky barrier (SB) at the IGZO-graphene interface. Next, an ion gel/P(VDF-TrFE) gate dielectric layer (i.e., the retentive EDL) was spin-coated onto graphene. In the retentive EDL, the P(VDF-TrFE) layer served as an ion transport–mitigating layer. Last, an Al presynaptic terminal was deposited onto the retentive EDL (see the transfer and output characteristics of the resulting artificial synaptic device in fig. S3). Briefly, presynaptic voltage (*V*_pre_) was applied at the Al presynaptic terminal, and postsynaptic current (PSC) (*I*_post_) was transmitted to the graphene postsynaptic terminal after signal processing by the retentive EDL. Here, *V*_pre_ and *I*_post_ correspond to presynaptic spike and postsynaptic signal in Fig. 2A. The detailed working mechanism of the AS is depicted in Fig. 2C. At low voltages (*V*_pre_ < 3 V), the transport of [EMIM]^+^ and [TFSI]^−^ was mostly limited to within the P(VDF-HFP) matrix, and their transport through the adjacent P(VDF-TrFE) layer (i.e., the ion transport–mitigating layer) was restricted. In contrast, at high voltages (*V*_pre_ ≥ 3 V), the electric field was high enough for [EMIM]^+^ and [TFSI]^−^ to readily penetrate into the P(VDF-TrFE) layer. Because the as-penetrated ions could be released only from the P(VDF-TrFE) layer over time, the formation/deformation behavior of the EDL was retentive. To verify this operation mechanism, we used a testbed device comprising the ion gel and ion transport–mitigating layers sandwiched between Al and ITO electrodes (fig. S4). Electrochemical impedance spectroscopy (EIS) analysis was performed on the testbed device, as shown in Fig. 2D. It should be noted that the ITO electrode was selected, which is vulnerable to faradic reactions under contact with ionic liquids. The Nyquist plot revealed distinct behaviors at different operation voltages. At low voltages below 3 V, a tilted straight line was observed in the plot, indicating that the testbed device behaved as a capacitor with some degree of nonideality. Because the transport of [EMIM]^+^ and [TFSI]^−^ was considered to occur within the P(VDF-HFP) matrix only and not through the adjacent P(VDF-TrFE) layer at these voltages, the retentive EDL could be simply considered as two capacitors connected in series: an EDL capacitor (the ion gel layer) and a dielectric capacitor (the ion-free ion transport–mitigating layer). At voltages above 3 V, a semicircle was observed in the Nyquist plot, indicating that the testbed device could be considered to be equivalent to a capacitor and a resistor connected in parallel. The appearance of the resistor component (i.e., the charge transfer resistance) under these voltage conditions is attributed to the ions that penetrated into the P(VDF-TrFE) layer, which ended up making direct contact with the ITO electrode. As mentioned above, ITO is vulnerable to electrochemical degradation when in contact with [EMIM][TFSI]. As the voltage increased, more ions could penetrate into the P(VDF-TrFE) layer, which led to a smaller charge transfer resistance with a smaller semicircle in the Nyquist plot.

These ion transport properties in the retentive EDL influence the work function of graphene (*W*_gr_). To visualize the modulation and retention of *W*_gr_ by ion movement in the retentive EDL, we performed Kelvin probe force microscopy (KPFM) measurements, as shown in Fig. 2E. The contact potential difference (*V*_CPD_) between graphene and the KPFM tip was measured at various voltages (0 to 5 V in 1-V steps) applied to the ITO electrode. *W*_gr_ was calculated as *W*_gr_ = *W*_tip_ − *eV*_CPD_, where *W*_tip_ is the work function of the tip (4.2 eV). Figure 2F shows the KPFM images and corresponding *W*_gr_ values at various voltages. The graphene work function on the P(VDF-TrFE) layer was estimated to be 4.58 eV at *V*_pre_ = 0 V, and it was found to be lower at a higher *V*_pre_ (4.36 eV at *V*_pre_ = 5 V). The electric field generated by ion movement in the retentive EDL influenced the charge density of graphene. As a result, the variation in charge density caused a notable change in the work function and modulation of the SB height between graphene and the IGZO layer. (fig. S5). The ion movement in the ion transport–mitigating layer also affected the retention property of *W*_gr_. To confirm this, *W*_gr_ was monitored as a function of time after application of 3-, 4-, and 5-V voltage pulses at the ITO electrode. Figure 2G shows the changes in *W*_gr_ with time (also see fig. S6), which reveals that *W*_gr_ decreased gradually with time. The time constant (τ) of the decay curves increased with an increase in the applied *V*_pre_ (17.9, 20.1, and 26.1 s at 3, 4, and 5 V, respectively). A larger τ implies that the ion transport–mitigating layer maintained the decrease in the work function for a long time and thereby enhanced the synaptic connections for prolonged periods of time. It should be noted that the work function did not return to its initial value (4.58 eV) even after 60 s.

On the basis of the above-described synapse facilitation mechanism of the retentive EDL, the LTP behavior of the PSC (denoted by *I*_post_) was examined as a function of the electrical pulse input (3, 4, and 5 V under λ_pre_ = 25 ms) (Fig. 2H). When the *V*_pre_ pulse was applied to the AS, *I*_post_ increased abruptly and then decayed, which is analogous to STP. After the decay of the *I*_post_, LTP characteristics showed retention of *I*_post_. This behavior became prominent at higher *V*_pre_. A higher *I*_post_ was measured with at higher *V*_pre_ because of the enhanced synaptic connections (fig. S7). For comparison, the LTP behavior of a device with only the ion gel, i.e., without the P(VDF-TrFE) layer, was also analyzed to confirm the role of this layer as the ion transport–mitigating layer. When *V*_pre_ of 3 V was applied to this device, a rapid decrease occurred in *I*_post_, which resulted in negligible LTP characteristics. Therefore, it is obvious that the ion transport–mitigating layer plays a key role in the generation of LTP characteristics. The LTP characteristics could also be controlled by tuning of the pulse width (λ_pre_) of presynaptic spikes (fig. S8). To achieve depression of *I*_post_, we applied negative *V*_pre_ (−3, −4, and −5 V), which induced STD and LTD behaviors as shown in fig. S9. The effect of the ion transport–mitigating layer could also be observed from the enhanced retention of *I*_post_ under the application of consecutive pulses. Figure 2I and fig. S10 show the application of paired pulses within a short time interval (Δ*t*) and the subsequent increase in *I*_post_. The increased *I*_post_ value at 2 s after application of the first pulse and the second (consecutive) pulse is denoted as *I*_post1_ and *I*_post2_, respectively. Figure 2J shows the ratio of *I*_post2_ to *I*_post1_ (*I*_post2_/*I*_post1_) as a function of Δ*t*, where this ratio is an indicator of enhancement of synaptic connections. *I*_post2_/*I*_post1_ was high at small values of Δ*t* because a strengthened synaptic connection by the first pulse was still dominant. At larger values of Δ*t*, *I*_post2_/*I*_post1_ decreased but remained at a level above a certain value owing to the weaker strengthening effect of the first pulse. Meanwhile, the ion gel–only device had an *I*_post2_/*I*_post1_ value close to one regardless of Δ*t*, which supported our observation that the ion transport–mitigating layer contributed notably to maintaining synaptic connections (fig. S11).

### Artificial nervous system with AS circuit and AN circuit

**Fig. 3:**
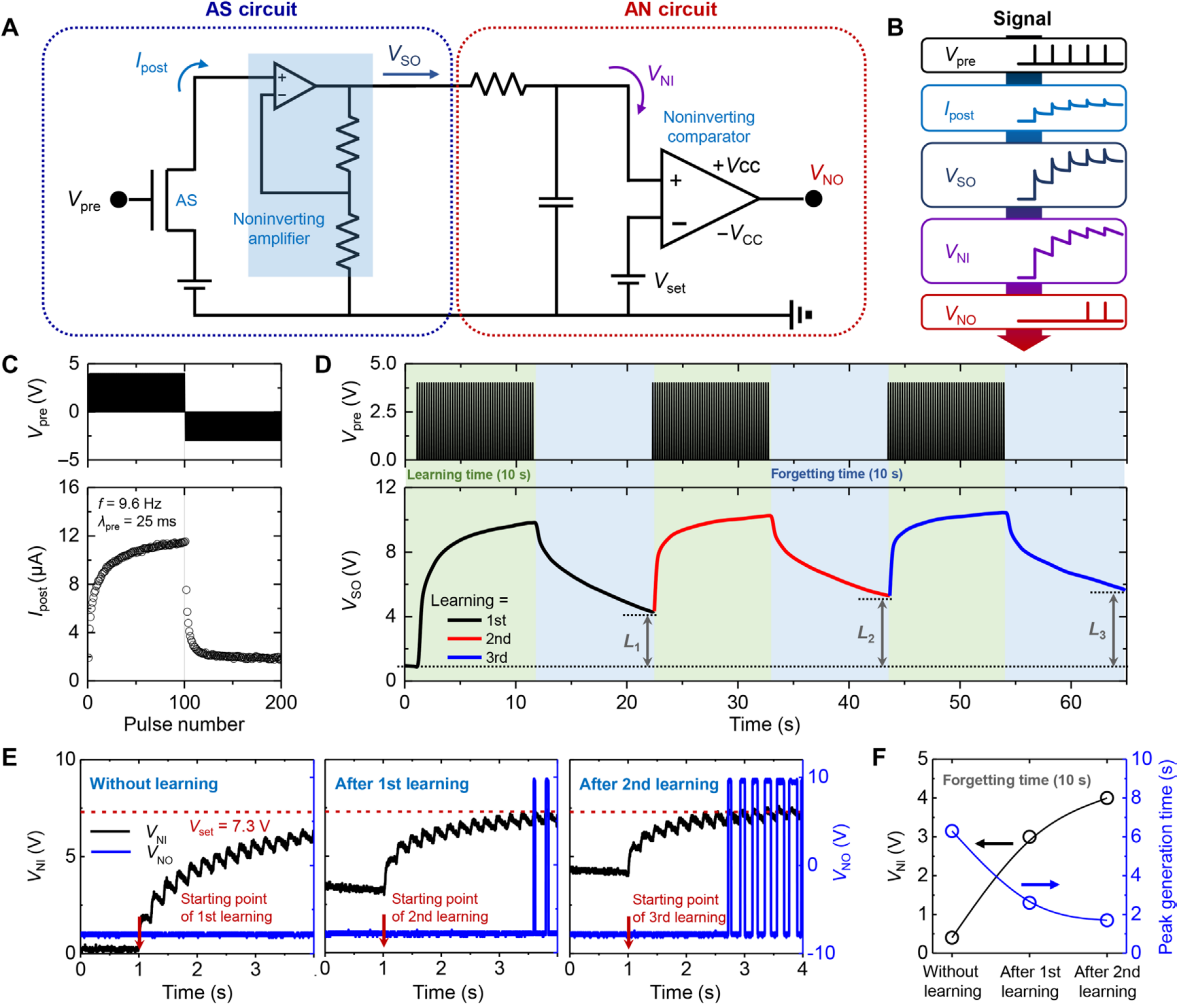
Artificial nervous system with AS circuit and AN circuit. (**A**) Circuit diagram of artificial nervous system incorporated with AS circuit and AN circuit. (**B**) Signal transmission process in artificial nervous system. (**C**) LTP/LTD characteristics of AS induced by application of consecutive *V*_pre_ pulses. (**D**) Learning process of AS circuit, which involves application of positive *V*_pre_ pulses (i.e., learning periods) alternating with forgetting periods (10 s each). *L*_1_, *L*_2_, and *L*_3_ are defined as the *V*_SO_ increments from its value in the initial state to those after each forgetting period of 10 s. (**E**) Iterative learning process of AS circuit. (**F**) Plots of *V*_NI_ and peak generation time for different learning processes (i.e., without learning, after first learning process, and after second learning process).

A firing component capable of processing the strengthened signal in the AS is required for complete realization of the signal transmission system. Therefore, an AN circuit capable of analyzing and interpreting this signal was designed. Figure 3A shows the incorporation of the AS and AN circuits into the artificial nervous system. The AS circuit, which is aimed at amplifying a weak AS signal, is composed of the AS and a noninverting amplifier (×10^3^). The AN circuit is composed of a resistor-capacitor (RC) circuit and a noninverting comparator to mimic the leaky integrate-and-fire (LIF) neuron model. The LIF model states that the neuronal action potential (*V*_NO_) is generated by leaky accumulation of the synaptic output voltage (*V*_SO_). In the AN circuit, leaky accumulation occurs through the RC circuit and leads to the generation of *V*_NO_ by the comparator. Figure 3B shows the signal transmission process in detail. First, a *V*_pre_ pulse train is applied to the AS, which is subsequently converted into *I*_post_. Next, *I*_post_ flows into the noninverting amplifier to magnify the input signal and is converted into *V*_SO_. In the AN circuit, *V*_SO_ is converted into a neuron input voltage (*V*_NI_) through charging and discharging of the RC circuit. It should be noted that *V*_NI_ shows leaky integration with a peak and trough owing to the STP and LTP behaviors of the AS. Last, *V*_NI_ is delivered to the comparator to determine whether it exceeds the threshold voltage. When the accumulated *V*_NI_ is lower than the set voltage (*V*_set_), the comparator outputs −*V*_CC_ as *V*_NO_. Conversely, if *V*_NI_ is higher than *V*_set_, then the comparator outputs +*V*_CC_ as *V*_NO_. Thus, it is possible to generate a *V*_NO_ pulse train by setting *V*_set_ to an appropriate value, which is referred to as the firing process in the LIF model.

To verify the signal flow described in Fig. 3B, we measured the output signals of the AS and AN circuits. First, 100 potentiation pulses (+4 V) followed by 100 depression pulses (−3 V) were consecutively applied as *V*_pre_ at a frequency of 9.6 Hz and a pulse width of 25 ms (Fig. 3C, top). The LTP and LTD properties were observed through the application of the consecutive pulses, as shown in Fig. 3C (bottom) and fig. S12 (stability test). During LTP, application of repeated potentiation pulses to the retentive EDL–based AS strengthened the synaptic connections. In contrast, application of repeated depression pulses led to a decrease in *I*_post_ and its return to the initial state, which is characterized as LTD. Figure 3D shows the process of learning in the AS by the utilization of the LTP characteristics. The top panel shows three *V*_pre_ pulse trains for learning, wherein learning periods were alternated with forgetting periods (10 s each). Each *V*_pre_ pulse train corresponds to the first 50 potentiation pulses depicted in Fig. 3C. Application of the first pulse train led to an increase in *V*_SO_, which resulted in facilitation of synaptic connections. During the forgetting period, *V*_SO_ decreased gradually with time, which indicates weakened synaptic connections. Here, *L* is defined as the increase in *V*_SO_ from its value in the initial state to that after a forgetting time of 10 s, the value of *L* increased from 4.3 V (after the first learning process) to 5.6 V (after the third learning process), as shown in fig. S13. That is, the increase in *V*_SO_ was maintained over the long term, despite the forgetting periods in between the learning periods. Furthermore, the unlearning process in the artificial nervous system is demonstrated, as shown in fig. S14. By applying negative *V*_pre_ at the end of the second and third learning processes, the *L*_1_ value was maintained after the second and third learning processes. Figure 3E depicts an iterative learning process with *V*_NI_ and *V*_NO_ over time. *V*_NO_ was generated when *V*_NI_ exceeded *V*_set_ (7.3 V). Before the learning process, the peak generation time of *V*_NO_ was 6.3 s. The peak generation time decreased to 1.7 s after the second learning process (Fig. 3F). In this manner, verification of the signal flow in the signal transmission system provided a clear validation of the learning ability of the artificial nervous system.

### Artificial stimulus-response system emulating human conscious response

**Fig. 4:**
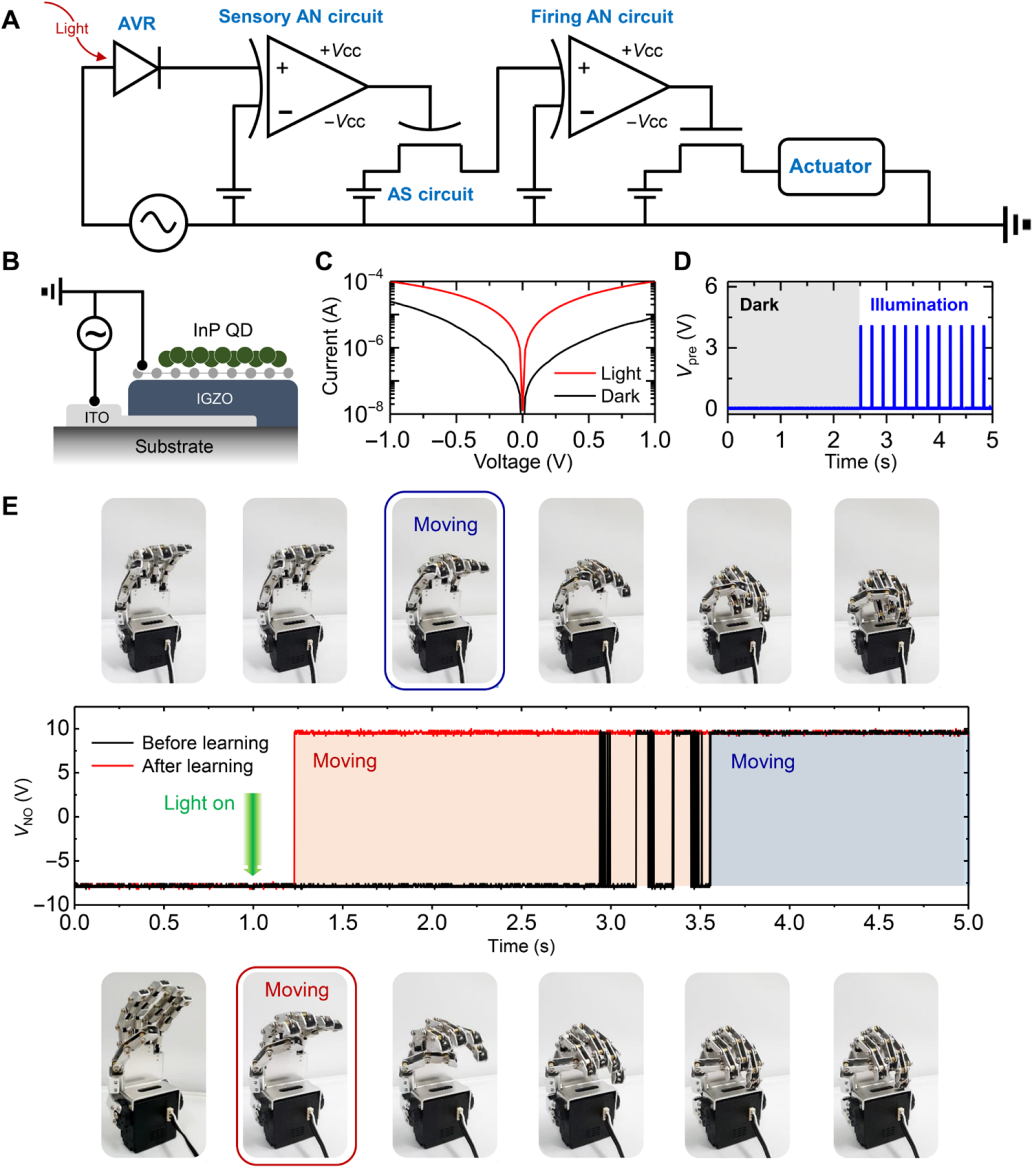
Artificial stimulus-response system emulating human conscious response. (**A**) Circuit diagram of artificial stimulus-response system with AVR, sensory AN circuit, AS circuit, firing AN circuit, and actuator. (**B**) Cross-sectional schematic of AVR with vertically stacked graphene/IGZO/ITO heterostructure. (**C**) Current-voltage characteristics of AVR under dark and illumination conditions (50 μW, 520 nm). (**D**) Dynamic response of *V*_pre_ generated by AVR and sensory AN circuit under dark and illumination conditions. (**E**) Real-time photographic images of robot hand and *V*_NO_ signals fired from firing AN circuit before and after learning. Photo credit: S. Kim [SKKU Advanced Institute of Nanotechnology (SAINT), Sungkyunkwan University].

Last, an artificial stimulus-response system was constructed as a proof of concept of its emulation of human conscious response. The stimulus and its response were set as depicted in Fig. 1D: The learning process causes the robot hand to promptly perform grabbing action after recognition of a visual signal. Figure 4A shows a schematic of the constructed system, which includes the AVR, sensory AN circuit, AS circuit, firing AN circuit, and actuator. The stimulus-response process begins with the absorption of visible light and the conversion of this stimulus into an electrical pulse by the AVR. Here, the incident visible light and the AVR are considered to correspond to, respectively, a visual stimulus and sensory receptor in the biological stimulus-response system. Figure 4B depicts the cross-sectional structure of the AVR. Here, the graphene/IGZO/ITO heterostructure functions as an SB diode. Under illumination, the InP QD layer absorbs light (fig. S15) to induce a positive photogating effect on graphene. The photogating effect was visualized through the KPFM measurement as shown in fig. S16. The photo-generated electrons were transferred to graphene while photo-generated holes remained in InP QDs. Because of the charge separation, the work function of the InP QD layer was increased, and the SB at the graphene-IGZO junction was reduced, which eventually led to an increase in the diode current as shown in fig. S17 (band diagram). The diode characteristics under light illumination are shown in Fig. 4C and fig. S18. The light stimulus is converted to *V*_pre_ through application of a voltage pulse input (*V*_pulse_) with an amplitude of 2 V (−1 and 1 V) and a frequency of 9.6 Hz to the AVR, which then enables control of the *V*_pre_ pulse train. As a result, the photocurrent change (from 8 to 100 μA at 1 V) is converted to a voltage change (from 8 to 100 mV, respectively). A signal exceeding the threshold value (60 mV) leads to fire operation of the sensory AN circuit (fig. S19). Figure 4D shows the dynamics of *V*_pre_ generated by the AVR and the sensory AN circuit under dark and illumination conditions. The sensory AN circuit, triggered by a signal exceeding the threshold value, generates *V*_pre_, which is then transmitted to the AS; this consequently results in the LTP characteristic of the AS. That is, the signal is transmitted to the AS only under light illumination, which induces a learning effect by strengthening the synaptic connections. After the signal processing by the AS circuit and firing AN circuit as depicted in Fig. 3, the transmitted signal lastly passes through the transistor and enters the robot hand.

Figure 4E shows the signals fired from the firing AN circuit and the consequent activation of the robot hand. The black and red lines indicate *V*_NO_ before and after learning, respectively. Here, the learning process was repeated three times; each learning process included a forgetting time of 10 s after exposure to the input light stimulus for 10 s. Initially, the artificial stimuli-response system with the robot hand was kept in the dark. After 1.0 s, light stimulus was applied to the robot hand both before and after learning. Before learning, the time required for activation of the robot hand was 2.56 s. However, after learning, the time interval between the application of the external signal input and the activation of the robot hand decreased notably to 0.23 s. This result confirms that our artificial neural system is able to emulate a conscious response and that it markedly improves the response time after learning from repeated light stimuli. Note that the pulse-based operation of our system enabled the lower energy consumption compared to all-CMOS–based circuit (table S1).

## DISCUSSION

To sum up, we designed an artificial stimulus-response system that successfully mimicked human conscious response using an AVR, an AS, AN circuits, and a robot hand. InP QDs and a retentive EDL were coated on top of a vertically stacked graphene/IGZO/ITO heterostructure serving as the base layer to realize the AVR and AS, respectively. In addition, an RC circuit and a comparator were used to construct sensory and firing AN circuits. These components of the artificial stimulus-response system were electrically integrated to induce a conscious response. Incident light signals were converted into electrical pulse signals by the AVR and sensory AN circuit. The pulse signals were then delivered to the AS and modulated synaptic connections by controlling the ion movement in the retentive EDL. Then, the postsynaptic signal entered the firing AN circuit. After several iterations of the learning process, the postsynaptic signal remained above a certain threshold level, as a result of which the robot hand moved faster in response to light stimulation. The proposed artificial stimulus-response system capable of conscious response provides new insights for research on artificial intelligence robotic systems.

## MATERIALS AND METHODS

### Device fabrication

For fabrication of the base layer of the devices, a 30-nm-thick ITO layer was deposited onto a glass substrate via radio frequency (RF) magnetron sputtering. The ITO layer was then patterned through conventional photolithography (AZ 5214E) and chemical etching [35 to 37 volume % (vol %) hydrochloric acid in distilled water] to form the drain electrodes. A 40-nm-thick IGZO layer was then deposited onto the patterned ITO electrodes by RF magnetron sputtering. The IGZO layer was then sintered at 300°C for 2 hours in an ambient atmosphere. The sintered IGZO layer was then patterned by photolithography and subsequent chemical etching with 3 vol % LCE-12 (Cyantek Co.) dissolved in distilled water. Separately, monolayer graphene was synthesized via chemical vapor deposition by a previously reported method. The synthesized graphene was then transferred using a poly(methyl methacrylate) supporting layer and patterned by photolithography and dry etching to form the source electrode. For fabrication of the AVR, InP QDs were synthesized through rapid injection of 0.001 M tris(dimethylamino)phosphine [(Me_2_N)_3_P] solution dissolved in oleylamine (OLA) into 0.001 M indium trichloride (InCl_3_) solution dissolved in OLA at 250°C. The reaction solution was then maintained for 1 hour. The synthesized InP QDs were washed with butanol and then redispersed in octane (200 mg/ml). The redispersed QD solution was spin-coated onto the base layer at 1000 rpm for 120 s. The ligand of the QDs was then replaced with ammonium thiocyanate (NH_4_SCN), specifically with 0.064 M NH_4_SCN solution dissolved in acetonitrile. For fabrication of the AS, 2 weight % solution of P(VDF-TrFE) (with a VDF:TrFE ratio of 70:30 mole percent; Arkema Inc.) dissolved in *N*,*N*-dimethylformamide was prepared, and the resultant P(VDF-TrFE) solution was spin-coated onto the base layer at 2000 rpm for 40 s. The P(VDF-TrFE) layer was then annealed at 140°C for 2 hours under ambient conditions. An ion gel composed of P(VDF-HFP) and [EMIM][TFSI] ionic liquid in a weight ratio of 1:4 was spin-coated onto the P(VDF-TrFE) layer at 3000 rpm for 40 s. The ion gel/P(VDF-TrFE) gate dielectric layer served as the retentive EDL. Last, the Al gate electrode was thermally deposited onto the retentive EDL through a shadow mask.

### Characterization

EIS was performed using an M204 multi-potentiostat (Autolab) under vacuum conditions. The work function of graphene was measured by KPFM (Park Systems NX10 atomic force microscope) under ambient conditions. The electrical properties of the vertical SB–based synaptic device were measured using a Keithley 4200A-SCS unit under vacuum conditions.

### Actuation of robotic hand

The robotic hands (QDS-15RO) and controlling unit (6 Channel Digital Servo Controller) were purchased in Lobot Robot, China. All fingers were actuated together by single input from the controlling unit (rated voltage, 5.8 to 8 V). The transistor (R6030KNX, ROHM Semiconductor) was driven by *V*_NO_ to filter the supply voltage (8 V) of the controlling unit.

## SUPPLEMENTARY MATERIALS

Supplementary material for this article is available at http://advances.sciencemag.org/cgi/content/full/7/15/eabe3996/DC1
